# Miliary Tuberculosis in an Elderly Woman: A Diagnostic Challenge in the Absence of Immunosuppression

**DOI:** 10.7759/cureus.101741

**Published:** 2026-01-17

**Authors:** Iker F Garcia Contreras, César A Vega López, Carlos A Gómez Núñez, Ana G Mazier Arita, Alexandra Marquez Carmona

**Affiliations:** 1 Internal Medicine, Hospital Angeles del Pedregal, Mexico City, MEX; 2 Internal Medicine, Hospital Angeles Pedregal, Mexico city, MEX

**Keywords:** case report, diagnostic challenge, elderly, immune senescence, miliary tuberculosis, mycobacterium tuberculosis

## Abstract

Currently, *Mycobacterium tuberculosis* (MTB) infection remains a major epidemiological health problem in developing countries. Although it primarily involves the lungs, this acid-fast bacillus can disseminate through lymphatic and hematogenous routes.

Over the past decades, the incidence of tuberculosis has significantly decreased, and vaccination campaigns have protected against severe forms such as meningeal and miliary tuberculosis. However, elderly populations remain vulnerable due to the absence of vaccination in earlier decades and the effects of immunosenescence. Extrapulmonary tuberculosis continues to pose a diagnostic challenge, particularly in patients with metabolic comorbidities. This report aims to describe the clinical and therapeutic characteristics of a case of miliary tuberculosis in an elderly patient with an insidious and nonspecific presentation.

## Introduction

Over the past decades, there has been a significant decline in severe forms of *Mycobacterium tuberculosis* (MTB) infection, such as miliary and meningeal tuberculosis, largely attributed to vaccination programs. Recent data estimate that approximately 23% of the global population is latently infected with MTB [1]. According to the World Health Organization [2] annual report, the highest incidence rates occur in Southeast Asia (45%), Africa (24%), and the Western Pacific (17%), whereas the Americas account for only 3.2% of global cases. Despite limited data regarding extrapulmonary manifestations, the prevalence of miliary tuberculosis among immunocompetent individuals has been reported to be less than 2% [3].

The pathophysiology involves lymphohematogenous dissemination of MTB from a pulmonary or extrapulmonary focus. Aging leads to detrimental alterations in the function and intercellular communication of pulmonary resident and peripheral immune cells, resulting in impaired innate and adaptive immune responses. This phenomenon, known as immunosenescence, is associated with increased susceptibility to infections such as miliary tuberculosis and with atypical or disseminated clinical presentations [4].

We report the case of an elderly female patient with insidious-onset miliary tuberculosis, diagnosed after extensive clinical and imaging evaluation, underscoring the importance of early recognition of this entity in geriatric populations presenting with atypical symptoms.

## Case presentation

An 80-year-old female presented to the emergency department with an acute onset of headache and confusion that began the previous night. Her family reported an oxygen saturation of 70% on room air at home; she denied prior use of supplemental oxygen. Directed questioning revealed an unintentional weight loss of approximately 11 kg over six months, intermittent low-grade fever predominantly in the mornings, and progressive exertional dyspnea accompanied by a dry cough over recent weeks. A private physician had prescribed empiric antibiotics and acetaminophen without clinical improvement, prompting hospital evaluation due to symptom persistence.

Her medical history included long-standing arterial hypertension, managed with telmisartan and lercanidipine, and a prior transient ischemic attack treated with an unspecified oral anticoagulant. She had no known history of tuberculosis exposure, recent travel, or immunosuppressive therapy.

Vital signs were as follows: blood pressure 145/87 mmHg, heart rate 76 beats/min, respiratory rate 24 breaths/min, temperature 37.6 °C, and oxygen saturation 85% on ambient air.

On physical examination, she was awake, alert, and oriented, though mildly bradypsychic. She appeared tachypneic, with bilateral reduction in chest expansion and mild intercostal retractions. Pulmonary auscultation revealed markedly diminished vesicular breath sounds in the mid-to-lower lung fields bilaterally, with dullness to percussion over the left hemithorax. No adventitious sounds were detected. Cardiac auscultation revealed a regular rhythm without murmurs. The abdomen was soft, non-tender, and non-distended, with normal bowel sounds. The extremities were thin but without signs of edema or neurovascular compromise.

**Figure 1:**
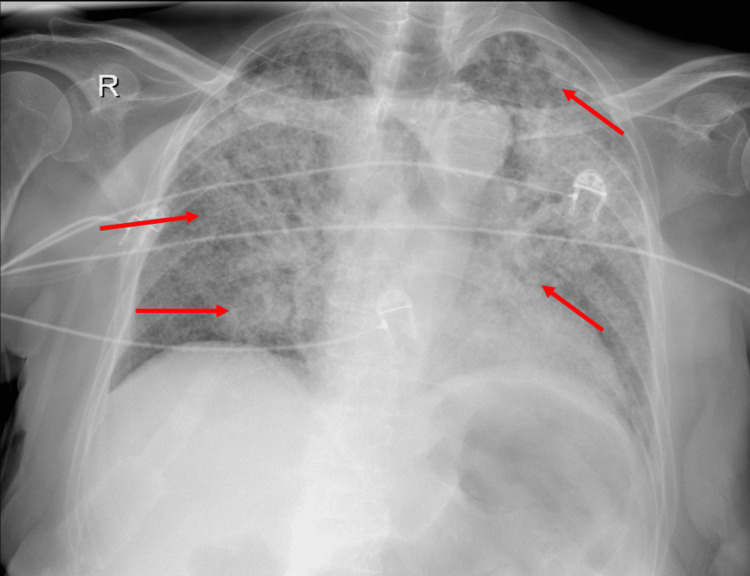
Chest radiograph: Diffuse bilateral increase in pulmonary density with patchy radio-opaque areas (red arrows) and visible air bronchograms, consistent with alveolar consolidation.

Routine laboratory tests (Table 1) showed mild lymphopenia, an elevated ESR, mildly increased transaminases, and a negative procalcitonin level. Initial chest radiography (Figure 1) revealed diffuse reticulonodular infiltrates.

**Table 1:** Baseline laboratory parameters, measured values, and reference intervals. AST, aspartate aminotransferase; ALT, alanine aminotransferase; ALP, alkaline phosphatase; ESR, erythrocyte sedimentation rate; MCV, mean corpuscular volume; HCH, mean corpuscular hemoglobin; RDW, red cell distribution width; WBC, white blood cell count

Test	Result	Reference interval
Hb	14.43 g/dL	12-16 g/dL
Hct	44%	37-48 %
MCV	90.5 fL	80-99 fL
HCH	29.7 pg	25-33 pg
RDW	16.5%	10%-15%
WBC	5.96 x 10^3^ mcL	3.80-11.20 x 10^3^ mcL
Neu	4.85 x 10^3^ mcL	1.50-7.80 x 10^3^ mcL
LYMPH	0.75 x 10^3^ mcL	0.80-4.50 x 10^3^ mcL
Plt	160 x 10^3^ mcL	130-400 x 10^3^ mcL
Glu	74 mg/dL	74-99 mg/dL
AST	76.5 U/L	5-34 U/L
ALT	58 U/L	0.0-55 U/L
ALP	100.4 U/L	40-150 U/L
ESR	41 mm/hour	0-15 mm/hour
Procalcitonin	0.13 ng/mL	0.00-0.50 ng/mL

**Figure 2:**
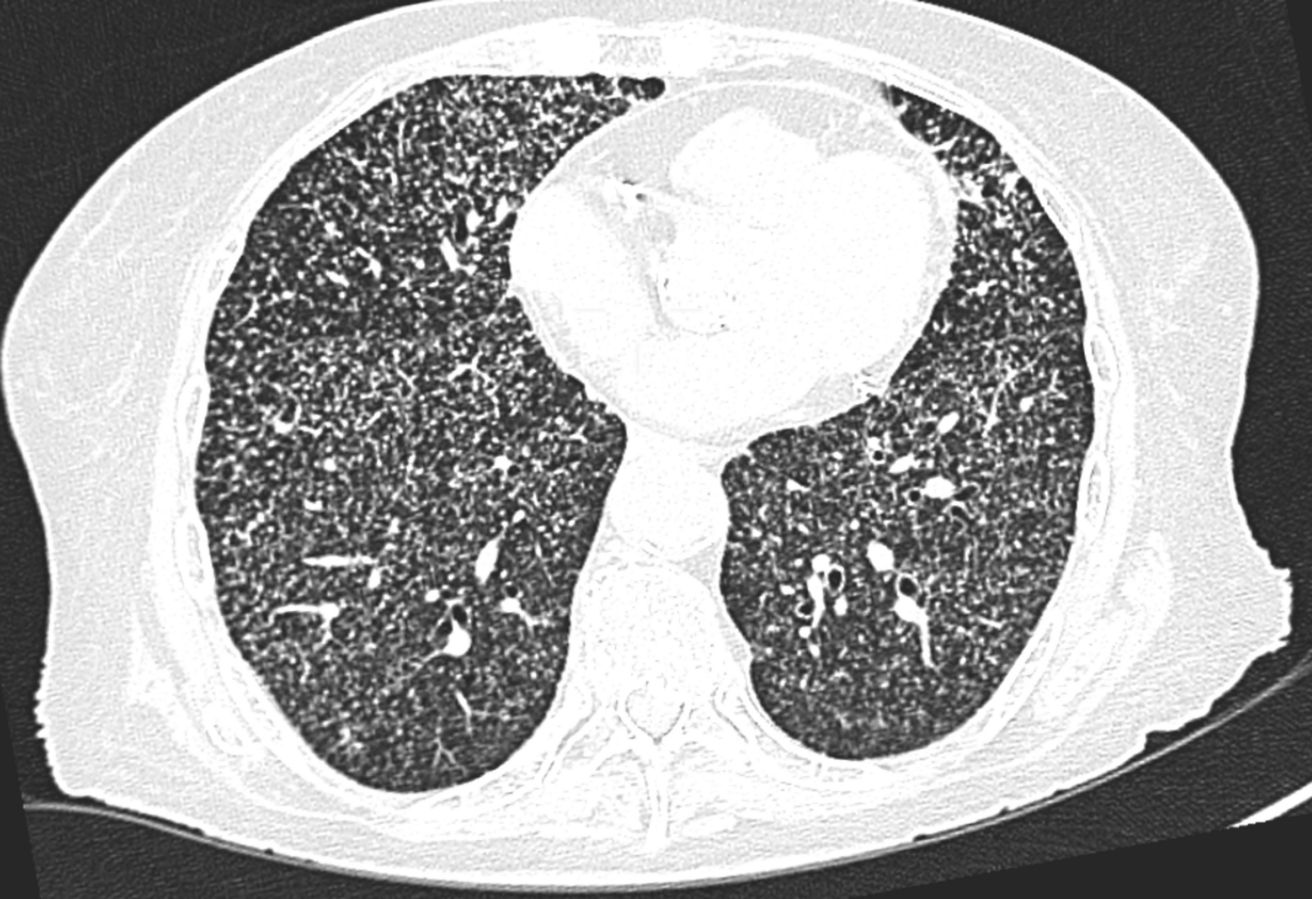
Axial high-resolution computed tomography (HRCT). Innumerable, randomly distributed micronodules (1-3 mm) throughout both lungs, predominantly in the middle and lower zones, consistent with miliary dissemination.

Continuing the diagnostic evaluation, a sputum culture and a multiplex polymerase chain reaction (PCR) assay using the BioFire FilmArray Pneumonia Panel (bioMérieux, Salt Lake City, UT) were performed. The panel was positive for Staphylococcus aureus (≥10⁷ copies/mL), with no mecA/C or MREJ resistance genes detected. However, considering the atypical presentation for community-acquired pneumonia and the patient’s clinical course, a high-resolution chest computed tomography (HRCT) scan was obtained (Figure 2), which revealed multiple diffusely distributed micronodules throughout both lungs.

During hospitalization, Ziehl-Neelsen staining and mycobacterial cultures from sputum samples were negative. As part of the extended diagnostic workup, a bronchoalveolar lavage cytology was performed from the left upper lobe, reporting only mild reactive changes of the respiratory epithelium associated with chronic inflammation. QuantiFERON-TB Plus (four tubes) was requested and yielded a positive result in the same tissue. PCR testing for MTB was also positive, with no rifampicin resistance detected.

The patient was initiated on standard first-line antituberculous therapy in accordance with international tuberculosis management guidelines, consisting of isoniazid (300 mg/day), rifampicin (600 mg/day), pyrazinamide (1,500 mg/day), and ethambutol (800 mg/day). After four weeks of treatment, she exhibited significant clinical improvement, with resolution of fever and an increase in oxygen saturation to 94% on room air. The total proposed duration of treatment was six months. 

## Discussion

Epidemiology

Miliary tuberculosis is an uncommon and severe form of extrapulmonary tuberculosis, representing approximately 1% of all active tuberculosis cases [5]. In a large retrospective multicenter analysis conducted in Turkey involving 263 adult patients diagnosed between 1981 and 2015, Mert et al. reported a higher prevalence in males (54%), with a mean age of 44 years and an overall one-year mortality rate of 17%. Most patients presented with nonspecific constitutional symptoms such as prolonged fever, asthenia, anorexia, and weight loss, and nearly half fulfilled criteria for fever of unknown origin. The most common predisposing factors included autoimmune diseases treated with steroids or immunosuppressants, type 2 diabetes mellitus, HIV infection, hematological malignancies, chronic kidney disease, and pregnancy [5]. Other studies have shown that miliary dissemination can occur in both immunocompetent and immunocompromised individuals, although its frequency increases substantially in the context of immunosuppression [3]. Despite advances in diagnosis and treatment, mortality associated with miliary tuberculosis has remained stable at approximately 15-30% over the past 25 years, highlighting persistent diagnostic delays and the complexity of its early recognition.

In recent years, epidemiological studies have demonstrated a demographic shift, with a marked increase in tuberculosis cases among the elderly population. Using data from a state-level tuberculosis prevalence survey in India, Giridharan et al. [6] reported an adjusted prevalence of microbiologically confirmed pulmonary tuberculosis of 482 cases per 100,000 inhabitants among individuals aged ≥60 years, almost three times higher than in adults under 60 years (adjusted prevalence ratio 2.99; 95% confidence interval (CI): 2.25-3.98). Factors associated with increased risk in this group included male sex, malnutrition, smoking, and a history of prior tuberculosis. These findings reflect the interplay between population aging, immunosenescence, and adverse socioeconomic conditions that limit access to timely diagnosis and treatment. Contemporary evidence suggests that population aging, together with persistent clinical and social determinants, may increasingly shape the epidemiological landscape of miliary tuberculosis.

Pathophysiology

Miliary tuberculosis represents the disseminated form of MTB infection, resulting from lymphohematogenous spread from a pulmonary or extrapulmonary focus to multiple organs, leading to the formation of numerous small granulomas distributed systemically [3]. The term *miliary *derives from the resemblance of the innumerable, uniformly distributed micronodules seen on imaging to millet seeds.

This process may occur during primary infection or through the reactivation of latent foci, particularly when host immune defenses fail. The ability of MTB to persist, replicate, and evade host immunity depends on a combination of structural and functional virulence factors that modulate pathogen-host interactions.

Key virulence determinants include components of the lipid-rich cell envelope, such as cord factor (trehalose-6,6’-dimycolate), lipoarabinomannan, and mycolic acids, which confer resistance to oxidative stress and facilitate survival within phagosomes. These lipids impair phagolysosomal maturation and inhibit the acidification required for bacterial killing, in addition to modulating the production of pro-inflammatory cytokines [7]. Secreted proteins such as Early Secreted Antigenic Target of 6 kDa (ESAT-6) and Culture Filtrate Protein of 10 kDa (CFP-10), components of the ESAT-6 Secretion System 1 (ESX-1), promote phagosomal membrane disruption and facilitate intercellular spread of MTB, ensuring its persistence within macrophages and subsequent hematogenous dissemination to highly perfused organs, including the liver, spleen, and bone marrow.

Control of infection depends primarily on cell-mediated immunity involving CD4+ and CD8+ T lymphocytes and activated macrophages. A Th1-mediated response characterized by the production of interferon-γ (IFN-γ) and tumor necrosis factor-α (TNF-α) is essential for granuloma formation and containment of the bacillus. In miliary tuberculosis, this balance is disrupted by an insufficient or Th2-skewed immune response, with predominance of IL-4 and IL-10, which suppress macrophage activation and favor intracellular replication of MTB [3]. Additionally, expansion of regulatory T cells (Treg, FoxP3+) at the site of infection contributes to local suppression of effector immunity, creating a permissive microenvironment for bacterial dissemination [3].

In older adults, the pathophysiology of miliary tuberculosis is further influenced by immunosenescence. Aging is associated with reduced T-cell functionality, diminished production of IFN-γ and TNF-α, and relative expansion of Treg populations that inhibit antimycobacterial activity [8]. Chronic low-grade inflammation (*inflammaging*) alters immune homeostasis and compromises the host’s ability to mount effective cellular responses against MTB. As a consequence, elderly patients often exhibit poorly organized granulomas and are at higher risk for hematogenous dissemination, explaining the increased frequency and severity of miliary tuberculosis in this population.

Histopathologically, miliarization consists of multiple uniform caseating foci in highly perfused organs, characterized by central necrosis and mononuclear infiltrates. In immunocompromised or elderly individuals, granuloma formation may be minimal or absent [3]. This pattern reflects the host’s inability to mount a contained immune response and represents the final outcome of the complex interplay between MTB virulence and host vulnerability.

Risk factors

Within the altered host-pathogen dynamic, several clinical and iatrogenic conditions act as predisposing factors for miliary tuberculosis. The most relevant risk factors include HIV/AIDS, malnutrition, diabetes mellitus, chronic kidney disease, autoimmune diseases treated with immunosuppressive agents, hematologic malignancies, pregnancy, and alcohol or tobacco use.

The coexistence of metabolic or infectious factors such as protein-calorie malnutrition and HIV infection further increases susceptibility by reducing the production of key pro-inflammatory cytokines (IFN-γ, IL-12, TNF-α) required for granuloma formation [3].

In their study, Mert et al. [5] reported that approximately 60% of patients had at least one predisposing condition. Diabetes mellitus was the most common comorbidity (24%), followed by prolonged corticosteroid use, solid or hematologic malignancies, and HIV infection. These factors not only increase disease incidence but are also associated with poorer outcomes and higher mortality. Importantly, the use of biologic agents targeting TNF-α (e.g., infliximab, adalimumab) has emerged as a significant risk factor, capable of triggering severe reactivation or miliary dissemination after only a few months of therapy.

Clinical presentation

Miliary tuberculosis occurs more frequently in children, accounting for 20%-40% of cases compared with 15%-30% in adults [9]. The disease may present with acute, subacute, or chronic manifestations, evolving over days, weeks, or months, with symptoms that vary according to the affected organ system. Fever is common, often with morning predominance, and is accompanied by nocturnal and evening sweating [9]. Respiratory distress syndrome occurs less frequently but is associated with worse outcomes in miliary tuberculosis [9].

Extrapulmonary dissemination may involve the lymphatic system, bones and joints, central nervous system (CNS), and adrenal glands. Hepatic involvement has been described, presenting with diffuse or localized right upper quadrant abdominal pain, nausea, vomiting, and diarrhea, and may rarely progress to liver failure [9].

CNS manifestations include tuberculous meningitis, tuberculomas, and transverse myelitis [9]. In immunocompetent individuals, tuberculous meningitis often presents with nonspecific symptoms such as fever, headache, somnolence, and confusion over two to three weeks. Meningeal signs, such as neck stiffness, may be absent. Progression of headache over one to two weeks without timely medical intervention may lead to obtundation, coma, or increased risk of death. Tuberculous meningitis increases the risk of stroke and seizures, and may present with focal neurological deficits, including monoplegia, hemiplegia, aphasia, or, less commonly, movement disorders such as choreoathetosis or hemiballismus. Ocular involvement may manifest as granulomatous uveitis. Approximately 50% of patients with tuberculous meningitis develop syndrome of inappropriate antidiuretic hormone secretion (SIADH) [9,10]. Hyponatremia is an important complication, and differentiating SIADH from cerebral salt-wasting syndrome may be challenging [11].

In spinal tuberculous meningitis, the acute presentation is characterized by progressive ascending paralysis and may involve cranial basal meningitis or other severe sequelae, whereas the subacute form may present with myeloradiculopathy, radicular pain, or progressive paraplegia or tetraplegia [9,12]. Up to 80% of patients may exhibit signs of elevated intracranial pressure, visual impairment, and cognitive decline, consistent with hydrocephalus [13].

Musculoskeletal involvement occurs in approximately 10% of extrapulmonary miliary tuberculosis cases. Symptoms typically begin gradually with back pain and tenderness; secondary complications include paresis, paraplegia, kyphosis, or scoliosis [9].

Immunocompromised patients or individuals with HIV and CD4+ counts <200 cells/mm³ often present with atypical manifestations (Table 2), including cutaneous lesions, intrathoracic lymphadenopathy, and poor response to tuberculin skin testing [9,12]. Cutaneous involvement is more frequent in patients with CD4+ counts <100 cells/μL and may present as macules, nodules, purpuric papules with vesicles, ulcerated plaques, or subcutaneous abscesses [12].

**Table 2:** Summary of clinical manifestations according to the affected organ and immunocompromised status in patients with miliary tuberculosis infection. Adapted from [1,2,4,6]. ALP, alkaline phosphatase; CT, computed tomography; MRI, magnetic resonance imaging

Involved organ or system	Immunocompromised status/frequency in HIV	Clinic presentation	Radiology findings	Prognosis
Pulmonary	High frequency in both immunocompetent and immunocompromised patients	Prolonged fever, dry cough, progressive dyspnea, weight loss	Chest X-ray: bilateral diffuse micronodular pattern (*millet seed* appearance). CT scan: uniformly distributed 1-3 mm nodules.	Poor without treatment; favorable when antituberculous therapy is initiated early
Central nervous system (meningeal/tuberculomas)	Very frequent in HIV patients with CD4 <100 cells/μL	Headache, altered mental status, neck stiffness, seizures	CT or MRI: basal meningeal enhancement, hydrocephalus, hypointense lesions with ring enhancement (tuberculomas)	Severe; high mortality and neurological sequelae without early diagnosis
Hepatic	Common in immunocompromised patients; may coexist with pulmonary tuberculosis	Fever, hepatomegaly, elevated transaminases and ALP, mild jaundice	Ultrasound: enlarged liver, hypoechoic lesions; biopsy: caseating granulomas	Fair to poor; depends on the degree of hepatic involvement
Spleen	Frequent in advanced HIV or extensive dissemination	Fever, splenomegaly, anemia, pancytopenia	Ultrasound or CT: microabscesses or multiple hypodense lesions	Poor prognosis; risk of sepsis or splenic rupture
Bone marrow	Frequent in HIV with severe immunosuppression	Fever, anemia, leukopenia, thrombocytopenia (pancytopenia)	Bone marrow aspirate: granulomas or acid-fast bacilli	Poor prognosis if associated with multiorgan failure
Renal	Moderate in HIV; more common in chronic stages	Sterile hematuria, fever, flank pain, proteinuria	CT urography: cavitations, calcifications, or ureteral stenosis	Variable prognosis; depends on early diagnosis
Peritoneal/Gastrointestinal	More common in immunocompromised patients	Abdominal pain, ascites, chronic diarrhea, weight loss	CT scan: peritoneal thickening, mesenteric lymphadenopathy, free fluid	Fair prognosis; good treatment response if diagnosed early
Disseminated meningoencephalitic (generalized miliary)	Very frequent in patients with advanced HIV	Persistent fever, general deterioration, hepatosplenomegaly, and confusion	CT/MRI: multisystemic findings; chest X-ray with miliary pattern	Severe; high mortality if not treated promptly

Diagnosis

The diagnosis of miliary tuberculosis requires correlation of clinical history, physical examination findings, and laboratory and imaging studies. 

Laboratory Findings

Cell count abnormalities are often nonspecific and may include pancytopenia, leukopenia, or leukocytosis with lymphocytic predominance; thrombocytopenia or thrombocytosis may also be observed in some cases [9]. An association between miliary tuberculosis and hemophagocytic lymphohistiocytosis has been described in certain patients [9,14]. The most frequent abnormalities include elevated acute-phase reactants, increased erythrocyte sedimentation rate, and elevated C-reactive protein. When neurological involvement is suspected, findings may include hyponatremia, hyperbilirubinemia, hypoalbuminemia, and, in some cases, elevated alkaline phosphatase; hypocalcemia is less frequently reported [9].

Biomarkers in Serous Fluids

The tuberculin skin test has low sensitivity and specificity in miliary tuberculosis due to anergy; however, positive results may occur even in the absence of active disease. Complementary diagnostic tools include adenosine deaminase (ADA) and IFN-γ testing in pleural, ascitic, and pericardial fluids [10,15]. In immunosuppressed or critically ill patients, urinary lipoarabinomannan testing has proven useful for rapid diagnosis [16].

Microbiological and Molecular Tests

Among the available diagnostic tools, direct smear microscopy is considered a low-sensitivity test in cases of miliary and disseminated tuberculosis, with a prolonged detection window compared with culture, regarded as the gold standard. However, culture has a sensitivity of only 20%-30% and requires a long turnaround time, often exceeding two weeks [17].

The GeneXpert MTB/RIF assay is highly specific and useful for detecting rifampicin resistance, particularly in multidrug-resistant tuberculosis [10,15]. This test significantly reduces diagnostic time in immunocompromised patients or those with disseminated disease, as DNA amplification from pulmonary or extrapulmonary specimens accelerates the detection process and improves diagnostic accuracy.

Imaging Studies

The clinical status of the patient guides the selection of imaging studies; therefore, the presenting symptoms must correspond to the suspected organ involvement. Chest radiography is a valuable tool for both diagnosis and clinical evaluation. The classic pattern of miliary tuberculosis consists of diffuse bilateral reticulonodular lesions.

In hepatic involvement, ultrasonography is useful for identifying lesions such as cold hepatic or splenic abscesses, intra-abdominal lymphadenopathy, and for guiding diagnostic procedures such as paracentesis for subsequent histopathological analysis [15].

Computed tomography (CT) and magnetic resonance imaging (MRI) allow visualization of disease extent in extrapulmonary sites, including the liver, spleen, intestines, peritoneum, adrenal glands, and lymph nodes. On CT, miliary tuberculosis lesions appear as discrete hypodense nodules, while classical nodular lesions <2 mm are observed on lung windows [10,15]. MRI provides superior characterization of CNS involvement, accurately identifying the extent of tuberculomas and cold abscesses, which is valuable for therapeutic planning [10,15].

Treatment

General measures include preventing or correcting malnutrition when present, as it is associated with immune dysfunction [18].

The standard regimen used for pulmonary tuberculosis also applies to miliary tuberculosis. According to WHO guidelines, the recommended treatment consists of a six-month regimen: an intensive (bactericidal) two-month phase with isoniazid, rifampicin, pyrazinamide, and ethambutol, followed by a four-month continuation phase with isoniazid and rifampicin. In pediatric patients, immunocompromised individuals, those with a slow clinical response, or those with complicated miliary tuberculosis, including lymphadenitis or skeletal tuberculosis, longer treatment durations may be required. For miliary tuberculosis, a 12-month regimen is recommended [9]; for drug-sensitive organisms, six to nine months may be adequate, while meningitis typically requires 9-12 months, and skeletal involvement approximately nine months [9,18].

To prevent drug-resistant tuberculosis, several key principles must be considered. Rifampicin remains the cornerstone of therapy, and most treatment regimens in resistant disease require at least 18 months of administration. Ethambutol plays an important role in preventing rifampicin resistance when the organism is isoniazid-resistant, and it may be discontinued once rifampicin susceptibility has been confirmed. Additionally, pyrazinamide is administered during the first two months to shorten total treatment duration from nine to six months [18].

Medical and surgical interventions may be required for diagnosis or therapy. Examples include mechanical ventilation for acute respiratory distress syndrome (ARDS), abdominal surgery for small-bowel perforation, and ventriculoperitoneal shunt placement for hydrocephalus, thereby reducing complications of tuberculous meningitis. Lymph node enlargement or intracerebral tuberculomas may appear during treatment, warranting neurosurgical evaluation [9,18].

Treatment of miliary tuberculosis during pregnancy includes isoniazid, rifampicin, and ethambutol, along with pyridoxine supplementation (25 mg/day). After delivery, histopathological examination of the placenta is recommended. If dissemination is suspected, the newborn should undergo gastric aspirates for testing; tuberculin skin testing is typically delayed until six months of age due to limited neonatal immune response [18].

Prognosis

In most cases, prognosis improves once antituberculous therapy is initiated; however, delayed treatment (1-8 days) has been associated with significantly higher mortality [19]. Diagnostic delays are common, prompting recommendations for the early use of broad-spectrum empirical antibiotics in cases of uncertainty [18,19].

Mortality is higher among elderly patients due to decreased pulmonary epithelial cell function, increased comorbidity burden, and diagnostic delays related to cognitive impairment. Between 16% and 24% of patients develop ARDS; mortality among elderly patients with ARDS ranges from 33% to 100% [19]. Mechanical ventilation is often required, and mortality rates in miliary tuberculosis may reach 25%-30%, and up to 65% in critically ill cases [20]. Among patients who develop ARDS and die, survival rarely exceeds three months.

Additional factors associated with poor prognosis include immunodeficiency, diabetes, psychiatric disorders, renal dysfunction, elevated liver enzymes, malnutrition, thrombocytopenia, and high blood urea nitrogen, associated with increased three-month mortality [19]. Other adverse prognostic markers include hyponatremia, hypoalbuminemia, leukopenia, markedly elevated transaminases, cancer, organ transplantation, HIV infection, malnutrition, silicosis, chronic kidney disease, surgical procedures associated with dissemination, and extensive ground-glass opacities on imaging. The neutrophil-to-lymphocyte ratio is a marker of systemic inflammation and has been used as a predictor of mortality, especially in ARDS. Alcohol and tobacco use also worsen prognosis [18-20].

The relapse rate is approximately 0-4% with appropriate supervised therapy, although estimates vary across studies. Most relapses occur within the first 24 months after treatment completion [18].

Complications

Delayed treatment of miliary tuberculosis may lead to the following complications [9]: ARDS, multiple organ dysfunction syndrome (MODS), tuberculous empyema, pneumothorax and pneumomediastinum (air-leak syndromes), tuberculous pericardial effusion and pericarditis, immune reconstitution inflammatory syndrome in some people living with HIV infection, myocarditis, native and prosthetic valve endocarditis, intracardiac masses, and tuberculous meningitis with focal neurological deficits; meningeal involvement has been reported in up to 25% of cases [18].

Additional neurological complications [21] include tuberculomas, myelopathies, paraplegia, transverse myelitis, and, less frequently, vision loss [22].

Organ involvement (<5%) versus systemic dissemination (>90%) has been documented in autopsy studies, with lesions identified in the lungs, liver, spleen, and brain [18]. Renal failure, pericarditis, neurological deterioration, thrombocytopenia, and hyperbilirubinemia have shown borderline associations with elevated erythrocyte sedimentation rate [22].

## Conclusions

Miliary tuberculosis remains a challenging clinical entity because of its heterogeneous presentation, frequent diagnostic delays, and substantial risk of morbidity and mortality, particularly among immunocompromised and elderly populations. Although advances in molecular testing and imaging have improved early recognition, the disease continues to demand a high index of suspicion, especially in patients with nonspecific systemic symptoms or risk factors such as HIV infection, diabetes, chronic kidney disease, or exposure to immunosuppressive therapies. A clear understanding of the mechanisms that allow MTB to evade immune control, together with the impact of immunosenescence, underscores the importance of rapid evaluation and timely therapy. Integrating laboratory markers, serous fluid biomarkers, and modern molecular diagnostics into the diagnostic workflow can significantly shorten time to detection, improving patient outcomes. Likewise, early initiation of appropriate antituberculous treatment, supported by tailored therapeutic durations in complex cases such as meningitis or disseminated disease, remains essential to reducing complications and mortality.

Given the persistent global burden of tuberculosis and the growing number of older and immunocompromised individuals at risk, clinicians must remain vigilant to the possibility of miliary dissemination. Strengthening diagnostic capacity, promoting early recognition of atypical presentations, and ensuring equitable access to effective therapy are key strategies to mitigate the clinical impact of this severe form of tuberculosis. Ultimately, improved awareness and timely interventions offer the best opportunity to change the trajectory of a disease that continues to pose significant challenges across diverse healthcare settings.
